# Infected Haematoma Fistulating Through Adjacent Colorectal Anastomosis Mimicking an Anastomotic Leak

**DOI:** 10.7759/cureus.74136

**Published:** 2024-11-21

**Authors:** William Qian, Mark Romero

**Affiliations:** 1 General Surgery, Royal North Shore Hospital, Sydney, AUS; 2 Faculty of Medicine and Health, The University of Sydney, Sydney, AUS; 3 General Surgery, Port Macquarie Base Hospital, Port Macquarie, AUS

**Keywords:** altemeier procedure, anastomotic leak, colorectal, colorectal surgery, fistula, general surgery, infected haematoma, postoperative abscess, postoperative complication, surgery

## Abstract

Anastomotic leakage is a well-understood major complication of colorectal surgery and carries significant implications for patient morbidity and mortality. However, an infected collection fistulating through an otherwise healthy colorectal anastomosis can mimic an anastomotic leak and warrants different management to a primary anastomotic leak. Such a presentation is undocumented in the current literature. A 42-year-old man underwent an elective perineal rectosigmoidectomy (Altemeier’s procedure) for full-thickness rectal prolapse. Early postoperative computed tomography (CT) imaging demonstrated a large haematoma in the rectovesical pouch. He represented 13 days later with fevers and lower abdominal pain. CT revealed that the known haematoma had evolved into an infected collection. At this stage, there was an intact anastomosis on imaging with no evidence of fistula formation or an anastomotic leak. There was a four-day delay between diagnosis and drainage of the abscess. By then, the collection had developed gas and a subsequent CT fistulogram confirmed a fistula had formed between the colorectal anastomosis and abscess, resembling an anastomotic leak. The patient was treated successfully with a diverting ileostomy and percutaneous drainage. We present a unique case of an infected haematoma fistulating rapidly through an otherwise healthy colorectal anastomosis, mimicking an anastomotic leak 17 days after an Altemeier procedure.

## Introduction

Anastomotic leakage is defined as a leak of luminal contents from a surgical joint between two hollow viscera [1] and is one of the most serious complications of colorectal surgery. It carries significant implications for patient morbidity and mortality [2] and is typically evidenced by the formation of an abscess and faecal fistula adjacent to the anastomosis [3]. We herein discuss a rare case of an infected postoperative haematoma that had formed a fistula into the rectum through a recently formed colorectal anastomosis, mimicking an anastomotic leak. This occurred 17 days after an Altemeier procedure for full-thickness rectal prolapse. While this presentation resembled an anastomotic leak, its management differed, and such a case has not been documented previously in the literature. The patient was treated successfully with the formation of a diverting loop ileostomy and CT-guided percutaneous drainage.

## Case presentation

**Figure 1:**
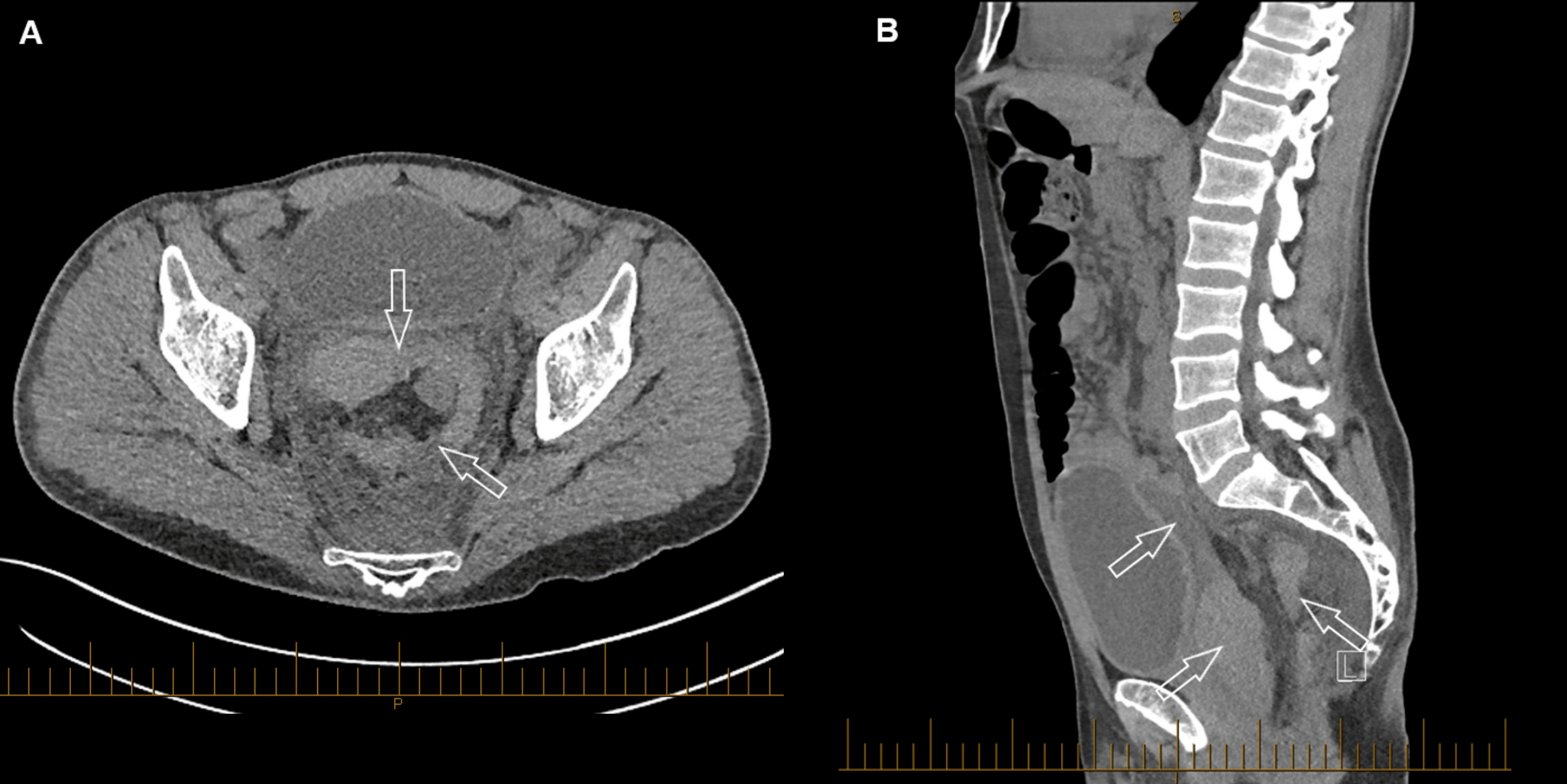
CT of the abdomen and pelvis two days after the Altemeier procedure. (A) axial, (B) sagittal, demonstrating a hyperdense lesion consistent with postoperative haematoma in the rectovesical pouch and extending to the presacral space. A slightly hyperdense haemoperitoneum in the pelvis is noted in (B).

A 42-year-old male underwent an elective Altemeier procedure for full-thickness rectal prolapse. This was on a background of schizophrenia and dyslipidaemia. The rectal prolapse was divided at the level of the rectosigmoid junction above the peritoneal reflection. Significant intraoperative bleeding was encountered, likely owing to branches of the superior and middle rectal arteries. This was adequately controlled with diathermy. A hand-sewn anastomosis between the remaining rectum and sigmoid colon was formed with 3-0 polydioxanone sutures (PDS) in two layers. On day two following the procedure, his routine blood count demonstrated a haemoglobin of 68, prompting CT angiography of the mesenteric and pelvic arteries. Whilst there was no active haemorrhage, the scan demonstrated a large hyperdense lesion consistent with a postoperative haematoma in the rectovesical pouch extending to the presacral space (Figure 1). There was also a moderate amount of slightly hyperdense-free fluid in the pelvis in keeping with the haemoperitoneum. He received a blood transfusion and was discharged home the next day, having recovered well.

**Figure 2:**
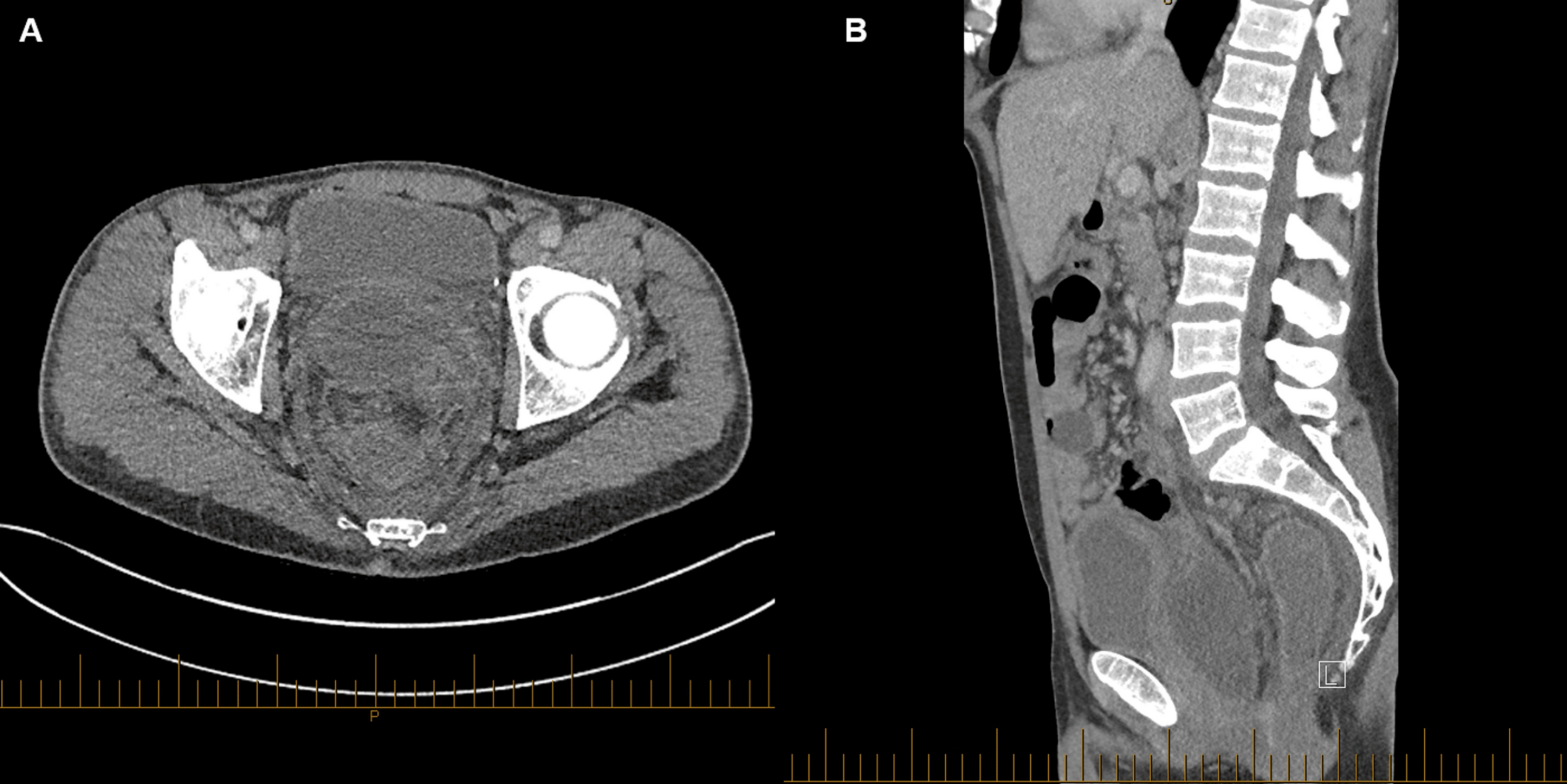
CT of the abdomen and pelvis 13 days after the Altemeier procedure. (A) axial, (B) sagittal, demonstrating an encapsulated infected collection evidenced by the thickened, enhancing peripheral wall and adjacent inflammatory fat stranding.

**Figure 3:**
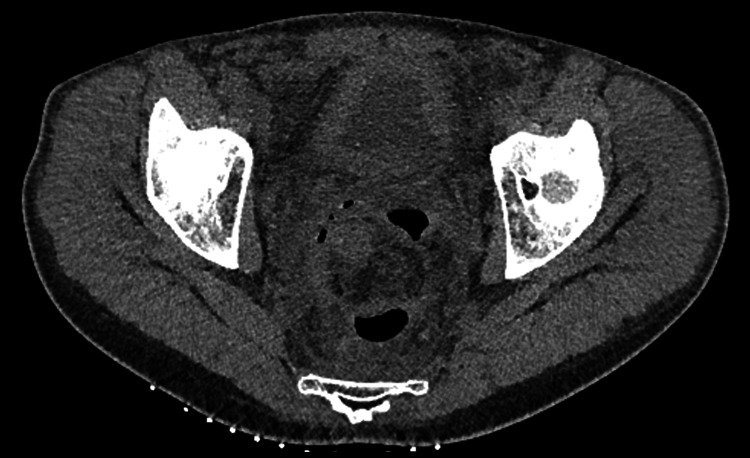
A planning pre-procedural CT of the abdomen and pelvis, just prior to drain insertion 17 days after the Altemeier procedure. Gas in the collection resembles an anastomotic leak (Axial).

The patient represented to the emergency department 10 days after discharge with fevers and lower abdominal pain. His blood biochemistry revealed an elevated C-reactive protein (CRP) of 256 mg/L. CT of the abdomen and pelvis demonstrated that the previously known large haematoma and haemoperitoneum had become replaced by an encapsulated fluid collection with a thickened, enhancing peripheral wall and adjacent inflammatory fat stranding (Figure 2). The diagnosis of a postoperative haematoma that had turned into an infected collection was made and the patient was admitted for intravenous antibiotics and interventional radiology (IR)-guided drainage of the abscess. Due to a temporary absence of proceduralists, the hospital’s usual IR service was not available until four days later. CT-guided drainage was planned for four days’ time and the patient was treated with intravenous ceftriaxone and metronidazole in the interim. Two days prior to drainage, the patient noted the passage of a moderate volume of per rectal blood. There were no other episodes prior to or following this. On the day of the drainage, a planning pre-procedural CT scan showed that the morphology of the abscess had changed markedly. The collection had reduced in size and there was now marked gas throughout the collection (Figure 3). These features were not present in prior imaging. CT-guided insertion of an eight-French pigtail drain into the perirectal collection was successful. Initially, minimal haemopurulent content was drained.

**Figure 4:**
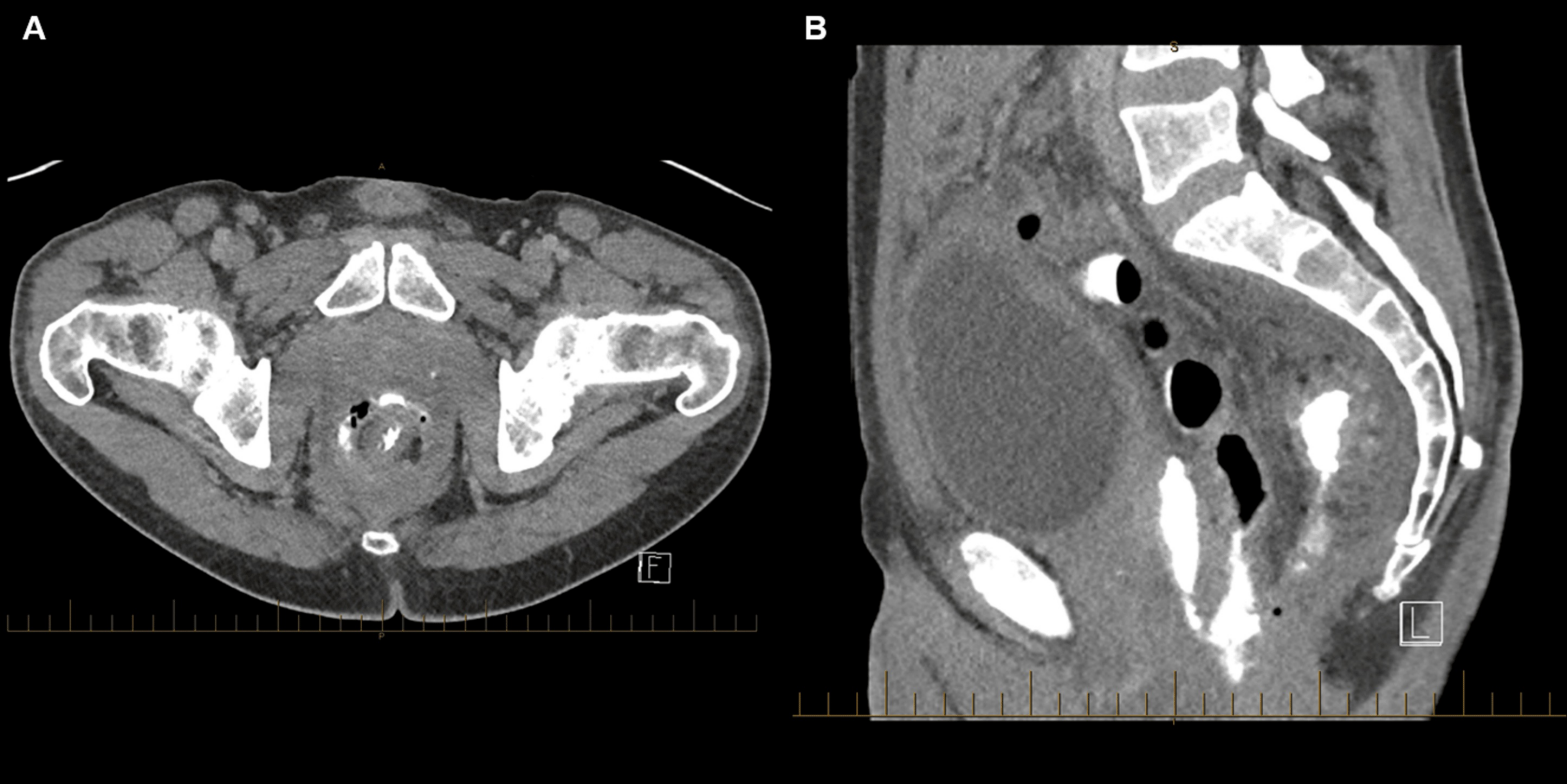
CT fistulogram of the abdomen and pelvis eighteen days after the Altemeier procedure. (A) axial, (B) sagittal. Contrast injected via the drain is visible in the collection and colon, confirming a fistula tract between the anastomosis and collection. The fistula tract is demonstrated in (B).

The next day, feculent content was noted in the drainage bag. A CT fistulogram with contrast injected through the drain confirmed the formation of a fistula between the colorectal anastomosis and perirectal abscess, with appearances resembling that of an anastomotic leak (Figure 4). There was no evidence of a fistula, anastomotic dehiscence, or gas within the abscess on initial presentation scans just five days prior, suggesting that this fistula had formed within the four days during which the patient was awaiting IR-guided drainage of the abscess. A diverting loop ileostomy was fashioned laparoscopically, and the patient had a slow inpatient recovery complicated by a prolonged ileus requiring a period of total parenteral nutrition (TPN). The drain was removed 10 days after insertion following a reduction in output and reassuring progress CT scan. The patient was discharged home after a total admission of twenty days. Five months later, he returned for an elective reversal of his loop ileostomy. A pre-operative flexible sigmoidoscopy revealed a healthy anastomosis, and he was discharged two days later following an uneventful operation and recovery. At a four-week follow-up, he was well with no new issues.

## Discussion

We report on a rare and previously undocumented scenario where an infected postoperative haematoma formed a fistula through an adjacent colorectal anastomosis, mimicking an anastomotic leak. Whilst an occult primary anastomotic leak cannot be entirely excluded, the clinical and radiological evidence heavily suggests that fistulisation through the anastomosis occurred secondary to the perirectal abscess in this case. The presence of a large postoperative haematoma was confirmed by CT on day two following the procedure. There was no evidence of a fistula or gas within the collection to suggest an anastomotic leak. This result was not unexpected given the bleeding encountered intraoperatively. On representation 13 days after the operation, CT suggested the haematoma had become infected, correlating with a raised CRP of 256 mg/L. However again, there was no evidence of fistulisation with the bowel or gas within the collection to suggest an anastomotic leak. The marked change in morphology of the abscess with the new presence of gas since the last scan four days prior suggests that the infected haematoma fistulated through the colorectal anastomosis during that time. This was confirmed on a CT fistulogram the next day demonstrating a fistula tract between the anterior surface of the colorectal anastomosis and the collection. Of note is the patient’s passage of a moderate volume of per rectal blood two days prior to the planned drainage of the infected haematoma. This is suggestive of the moment the abscess had fistulated through the colorectal anastomosis, spontaneously draining most of its contents, and correlates with the marked reduction in the size of the collection two days later on the pre-procedural CT (Figure 3).

Several factors may explain this sequence of events. Haematoma serves as an ideal medium for bacterial colonisation and proliferation. The large postoperative haematoma was seeded by bacteria during the Altemeier procedure due to the nature of its non-sterile, perianal approach. Worth noting is the high burden of surgical site infections (SSIs) following colorectal surgery. Whilst SSIs affect up to 5% of all surgical patients, they are the most common postoperative complication following colorectal surgery, with rates of approximately 20% being reported [4]. The increased pressure and inflammatory environment of an abscess impairs local tissue healing and integrity [5]. An important element in this case is the delayed percutaneous drainage due to the unavailability of IR proceduralists. The four-day delay between diagnosis and drainage of the abscess allowed for its inflammatory effects to facilitate the formation of a fistula tract between the abscess and rectum through the nearby, healing colorectal anastomosis. With evidence of an intact anastomosis and no fistula at the time of diagnosis of the abscess, timely drainage would have likely prevented this complication. This case demonstrates that indirect causes, such as local abscesses and infected collections, can rapidly fistulate through a colorectal anastomosis, mimicking an anastomotic leak after rectal surgery. Accordingly, surgeons should consider local abscesses as a modifiable risk factor for local faecal fistula formation, with prompt source control and drainage as viable preventative measures.

Colorectal anastomosis failure can markedly increase the burden experienced by a patient owing to increased length of hospitalisation, more interventions, and increased morbidity and mortality [6]. In this case, there was not a primary anastomotic leak, but rather a presentation mimicking one. While an anastomotic leak into the haematoma will infect it, an infected haematoma fistulating through a colorectal anastomosis will spontaneously drain it per rectum, as in this case. Given the known history of the infected haematoma, recent imaging confirming an intact anastomosis just days prior, and the lack of abdominal peritonism or clinical deterioration, the decision was made to treat this as an infected haematoma fistulating through the colorectal anastomosis. Had the patient experienced an anastomotic leak, management would have likely called for an emergency laparotomy, washout of the abdominal cavity, repair of the colorectal anastomosis, and formation of a diverting ileostomy. In this case, however, management of the fistulisation involved the unplanned formation of a diverting ileostomy, performed laparoscopically. In anastomotic leaks, early faecal diversion has been shown to reduce morbidity and mortality by decreasing the severity of leaks and subsequent complications [7]. In this case, the diversion was created to prevent faecal flow from maintaining communication with the abscess cavity, allowing for its collapse and the eventual sealing of the fistula between the abscess and colorectal anastomosis. The patient’s admission was then complicated by a prolonged ileus requiring a period of TPN, increasing the length of stay to twenty days. A subsequent operation and hospitalisation were required to reverse the ileostomy. These interventions and complications may have been avoided had the abscess been promptly drained once diagnosed, likely preventing the fistulisation through the anastomosis. This case demonstrates how subtle delays in care can majorly affect patient outcomes. Despite this, given that the anastomosis was otherwise intact and healthy, it healed without issue and the elective closure of the ileostomy was successful without complication. This, coupled with the patient’s complete recovery at follow-up, indicate that with appropriate intervention, patients can achieve favourable long-term outcomes.

## Conclusions

A rare case demonstrates that a local infected collection can fistulate rapidly through an otherwise healthy anastomosis following colorectal surgery, mimicking an anastomotic leak. This case offers rare insight into the natural history of such a presentation, as in most similar cases fistulisation may not have been observed due to timely management of the infected collection. The importance of expeditious drainage to prevent fistula formation and further complications is stressed. Surgeons should remain vigilant for evolving abscess formation and consider early intervention of postoperative collections, especially when they are adjacent to recently formed colorectal anastomoses.
